# Clinical outcomes of patients with pigmented villonodular synovitis of the shoulder after arthroscopic synovectomy

**DOI:** 10.1186/s12891-022-05978-3

**Published:** 2022-11-29

**Authors:** Yinghao Li, Lu Mei, Tao Li, Long Pang, Xin Tang, Jian Li

**Affiliations:** 1 Department of Orthopedics, Orthopedic Research Institute, West China Hospital, Sichuan University, No. 37 Guo Xue Xiang, Chengdu, 610041 Sichuan China; 2 West China School of Nursing, Sichuan University, Chengdu, China

**Keywords:** Arthroscopy, Synovectomy, Pigmented villonodular synovitis

## Abstract

**Background:**

Shoulder pigmented villonodular synovitis (PVNS) is a severe clinical condition, while few studies have focused on this situation due to its rarity. This study was to investigate the efficacy of arthroscopic treatment of patients diagnosed with shoulder PVNS.

**Methods:**

From Jan 1^st^, 2010 to Dec. 31^st^, 2019, 6 patients (5 females and 1 male) diagnosed with shoulder PVNS underwent arthroscopic synovectomy in our hospital and combined rotator cuff repair was performed in 3 of them. The outcomes of this study include Constant score, Visual Analogue Scale (VAS), University of California, Los Angeles (UCLA) score and American Shoulder and Elbow Surgeons (ASES) score. The data were retrieved from the patients’ medical records.

**Results:**

With a mean follow-up of 52.0 months (range, 28–92 months), the mean difference of Constant, VAS, UCLA and ASES scores were 27.83 ± 21.60, 2.83 ± 2.56, 11.67 ± 10.93 and 17.83 ± 25.35, respectively. Statistically significant improvements were detected in all the patient-reported outcomes except ASES score. One of the patients suffered from recurrence. Two patients suffered from mild complications after the surgeries while both of them achieved satisfactory recovery finally.

**Conclusion:**

Arthroscopic synovectomy in the setting of shoulder PVNS can improve patients’ function. A concurrent rotator cuff repair is recommended if it is needed. The conclusion still needs testifying by further high-quality research with larger sample size.

## Introduction

Pigmented villonodular synovitis (PVNS) is an unusual clinical condition characterized by benign tissue proliferation that involves synovium, tendon sheath, and bursa [1, 2]. The most affected joints are the knee and the hip [3]. The chief complaints include pain, swelling, stiffness, and limited range of motion in the affected joints [4]. Though a rare condition, this benign, highly proliferative neoplasm may result in massive joint destruction [1, 5]. What is more, patients reported by previous studies suggests that shoulder PVNS is often accompanied with massive rotator cuff tear [1, 2]. The poor rotator cuff condition would not only further impair the function of shoulder joint and seriously reduces the patients’ life quality, but also strongly influence the final clinical outcomes.

Shoulder PVNS is known to be extremely rare, accounting for about 2.0% to 8.0% in all PVNS cases [2, 5, 6]. Accordingly, few studies have focused on this situation, and, as a result, there is no accepted guideline for the treatment of PVNS as far as we know. Currently, the mainstay of treatment for PVNS for the vast majority of patients is arthroscopic and/or open surgery resecting the synovium [4]. Noailles et al [5] conducted a systematic review and suggested that arthroscopic excision of the lesions resulted in a satisfactory clinical outcome without recurrence in shoulder PVNS, but they also stated that it was difficult to recommend open debridement or arthroscopic synovectomy based on current evidence.

This retrospective case series aimed to report the clinical outcomes after arthroscopic synovectomy with/without rotator cuff repair in PVNS of the shoulder. We hypothesized that arthroscopic synovectomy would demonstrate good clinical outcomes in shoulder PVNS.

## Materials and methods

### Patients

The diagnosis of PVNS was considered when the patient presented the following symptoms before surgery: (1) pain and/or swelling in the affected joint, (2) locking, catching, and instability in the joint, (3) show an increase in the soft tissue density and/or lower bone density around the associated shoulder in plain radiographs, and (4) low signal intensity related to hemosiderin increase in nodule content and high signal intensity related to the increase in fat tissue in magnetic resonance imaging (MRI). The final diagnosis was made based on pathology of the tissue [7]. Patients who underwent open surgery, had bony lesions caused by other diseases or refused surgical treatment were excluded.

**Fig. 1:**
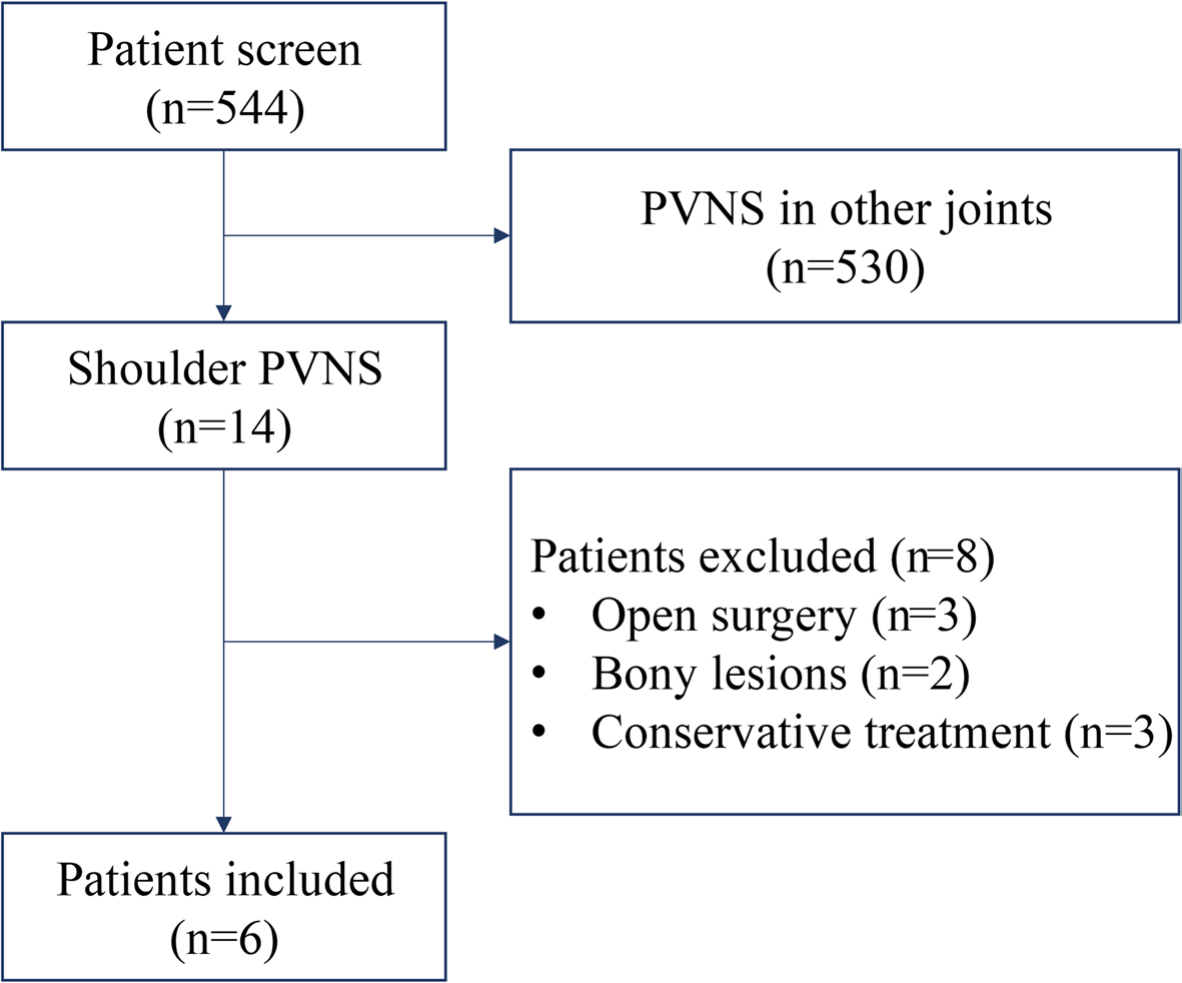
The flow diagram of patient inclusion. PVNS, pigmented villonodular synovitis

Based on symptoms, preoperative MRI and intraoperative pathology of tissue sampling, a total of 544 patients were diagnosed as having PVNS in our hospital from Jan 1^st^, 2010 to Dec. 31^st^, 2019. Fourteen (2.6%) of them suffered from shoulder PVNS and 6 underwent arthroscopic synovectomy (Figure 1). A rotator cuff repair was performed when necessary.

### Surgical technique

**Fig. 2:**
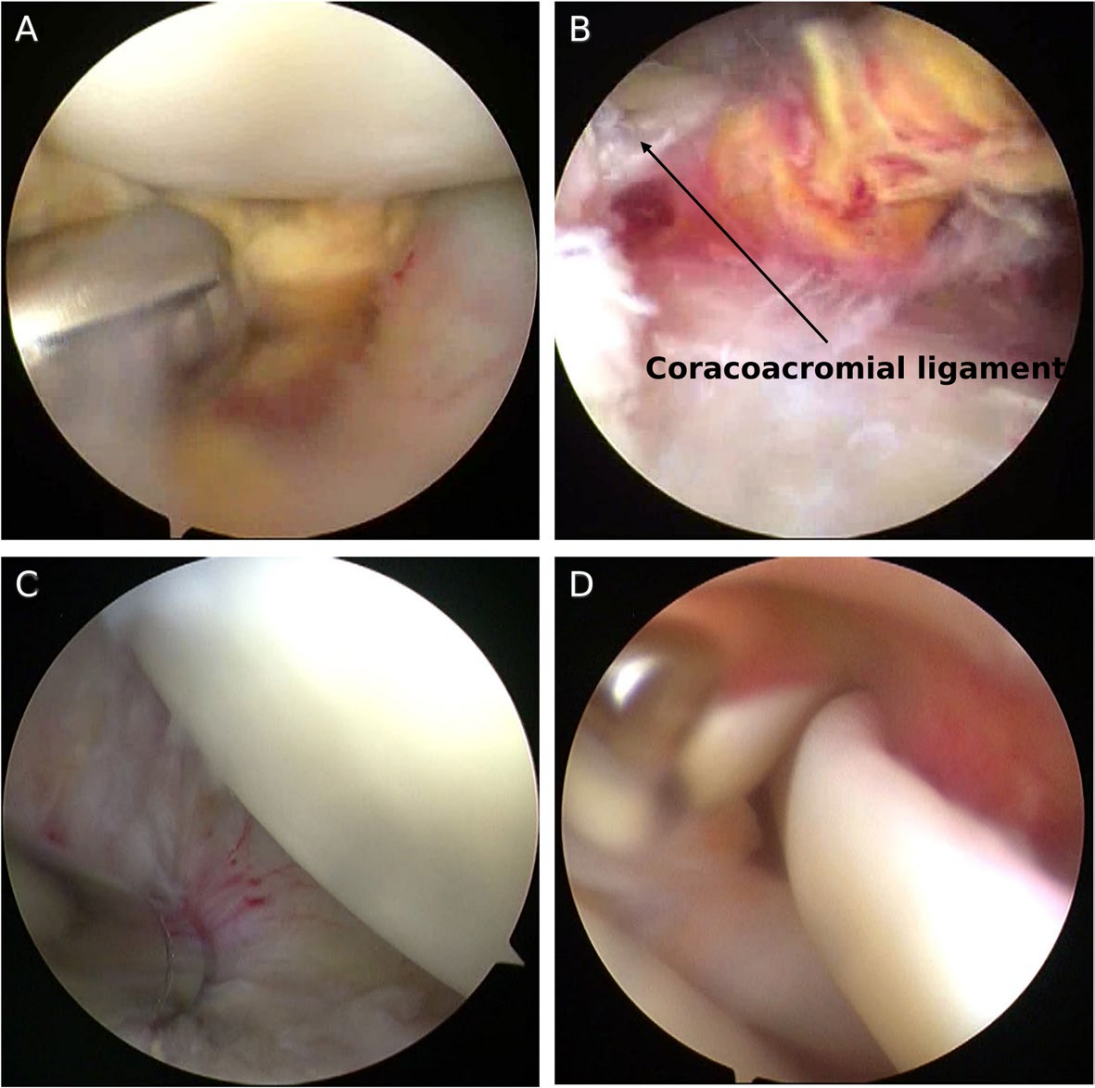
**A** lesion in the shoulder joint cavity (axillary capsule) was being removed by a shaver. **B** The huge lesion (yellow) under coracoacromial ligament was being pulled out by grasp. **C** The lesions and synovium in the axillary capsule region were totally resected. **D** The long head of biceps was dislocated and slack in case 1 after the removal of lesion

All the operations were performed by one senior surgeon. The patients were positioned in the lateral decubitus position and the affected shoulder was stretched. After general anesthesia, a standard posterior portal and an anterior central portal were established as described in the previous study [8]. An arthroscopic examination was then performed. The mass was removed using a grasper or shaver as well as the peripheral synovitis and the rest of the lesion (Figure 2A&B), and then a thorough synovectomy with debridement was performed with an arthroscopic shaver. Hemostasis was achieved by an ablation device prior to the completion of the procedure. Attempts were made in every surgery to completely resect the pathologic appearing tissue. The effect after removing the lesions and synovium is shown in figure 2C. Tissue samples of synovial tissue were stained with hematoxylin and eosin (HE) stain and examined under a light microscope by a senior pathologist.

After the lesions and hyperplastic synovium were removed, rotator cuff tears were found in 4 cases (case 1, case 3, case 4 and case 6) and the long head of biceps was dislocated and slack in case 1 (Figure 2D). Rotator cuff repair was performed in three patients (case 1,3 and 4). For case 1, after resection of the pathologic tissue and freshness of the insertion site on the greater tuberosity, rotator cuff was repaired by single row with three double loaded 4.5mm anchors(Smith&Nephew, USA) . For case 3, The long head of biceps was placed as the Chinese-way described to reconstruct the superior capsular [9]. Then rotator cuff was partially repaired by double row using suture bridge technique by two double loaded 4.5mm anchors and two one footprint ultra PK suture anchors(Smith&Nephew, USA) [10]. For case 4, a limited acromioplasty was performed firstly and then rotator cuff was repaired by two medial suture anchors and one footprint anchor(Smith&Nephew, USA)by suture bridge technique. The proximal ends of the torn tendons retracted back to the glenoid rim in case 6, which was regarded as irreparable rotator cuff tear. Rotator cuffs were intact in other cases.

### Postoperative rehabilitation

For patients who underwent both rotator cuff repair and synovectomy, the affected shoulder was immobilized in an arm sling for 2 weeks and the patients could only move their elbows and wrists in this period. After that, passive rehabilitation was applied under the protection of the brace while active exercise started from the 6th week. Resistance exercise was started 12 weeks after the surgery.

For patients who underwent synovectomy only, there was no strict limitation and active range of motion exercise could be performed immediately after the surgery.

### Outcomes and statistical analysis

The outcomes of this study include Constant score, Visual Analogue Scale (VAS), University of California, Los Angeles (UCLA) score and American Shoulder and Elbow Surgeons (ASES) score. The data were retrieved from the patients’ medical records. Minimal clinically important difference (MCID) was taken into consideration and the MCID of Constant score, UCLA score, ASES score and VAS were set at 6.3, 3.0, 15.2 and 1.4 respectively according to previous studies [11–13].

The data were analyzed with IBM SPSS Statistics (Version 25.0.0.1). The scores before the surgery and at the final survey were compared and tests for equal variances and normality were performed first. Paired-samples *t* test was adopted for variables with equal variances. Other way, independent *t* test was employed. A P value of < 0.05 was considered significant.

## Results

### Patients

**Fig. 3:**
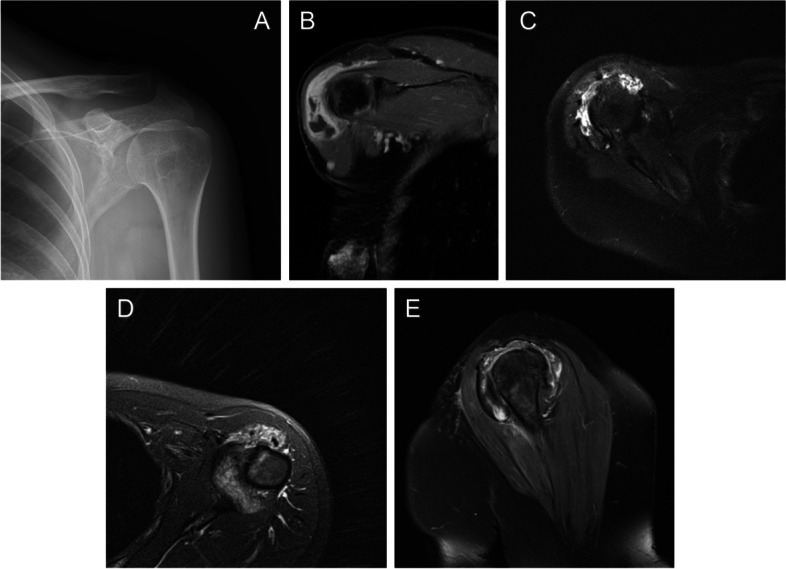
Radiological manifestations of shoulder PVNS. **A** Plain film shows low bone density in the front part of humeral head. **B**-**E** MRI shows low signal intensity related to hemosiderin increase in nodule content around shoulder cavity and high signal intensity related to the increase in fat tissue. PVNS, pigmented villonodular synovitis; MRI, magnetic resonance imaging

The baseline information of the patients is shown in Table 1. Five of the patients were female and the other one was male. The typical radiological manifestations of PVNS are shown in Figure 3. Their mean age at first surgery was 50.67 years (range, 30-65 years). The mean body mass index (BMI) was 22.29 kg/m^2^ (range, 18.03-28.76 kg/m^2^). The average follow-up time was 52.0 months (range, 28-92 months). Among them, 4 patients (case 1, case 3, case 4 and case 6) were diagnosed to have rotator cuff tear based on MRI.

**Table 1:** Baseline information of patients

Case No	Age at first surgery, y	Gender	BMI, kg/m^2^	Side	Size of rotator cuff tear	Type at first diagnosis	Radiotherapy after surgery	Follow-up, mo	Recurrence
1	45	F	19.72	Right^a^	Massive	Localized^b^	No	92	Yes
2	30	F	18.03	Left	NT	Localized	Yes	62	No
3	65	F	28.76	Right	Massive	Localized	No	44	No
4	62	F	23.56	Right	Massive	Localized	No	40	No
5	38	F	21.45	Right	NT	Localized	No	28	No
6	64	M	23.44	Right	Massive	Localized	No	46	No

*BMI* body mass index, *M* male, *F* female, *NT* no tear

^a^In the subsequent recurrence of the disease, the left shoulder was also affected

^b^In the subsequent recurrence of the disease, the type changed into diffuse

### Operative findings

**Fig. 4:**
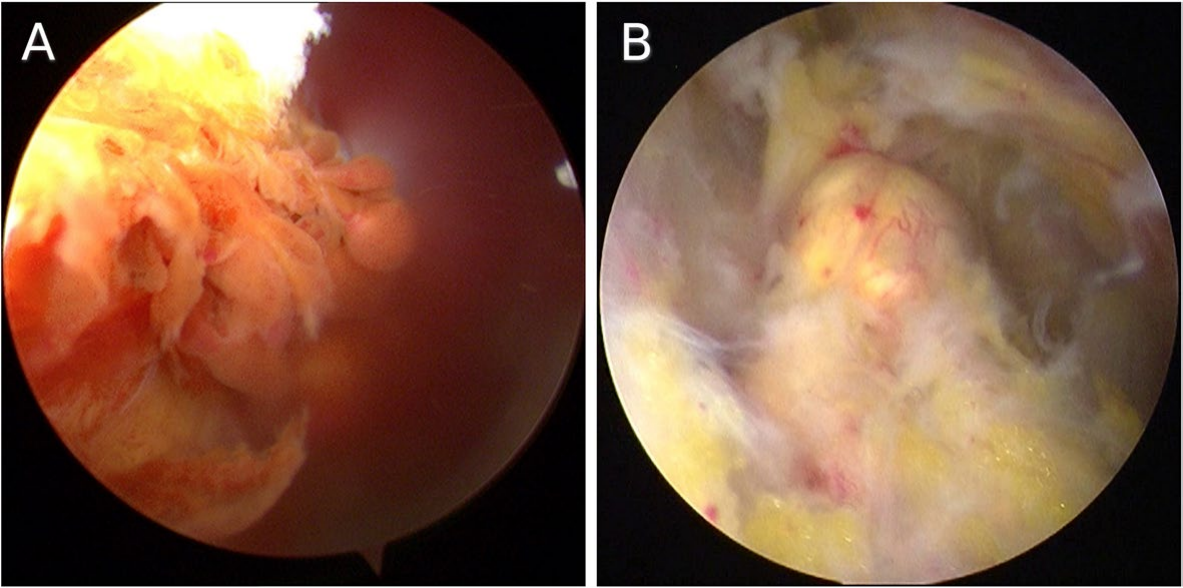
The operative findings during the surgery. **A** The reddish-brown synovium was hyperplastic with finger-like villous projections on it. **B** A lesion (yellow) in the subacromial space and angiogenesis was detected

All the shoulder joints were filled with brownish synovial fluid and hemosiderin. The reddish-brown synovium was hyperplastic with finger-like villous projections on it (Figure 4A). Typical villonodular lesions were found in 3 cases (case1, 2 and 5). In these 3 patients, more than 5 typical villonodular lesions were found within the rotator cuff and bursa mucosa in the articular cavity (case 1), around the tendon of subscapular muscle and long head of biceps (case 2), and in the subacromial space (case 5) (Figure 4B). Most of the nodules were small except the one found in case 2, whose size was about 7cm × 4cm × 2cm.

### Pathologic findings

**Fig. 5:**
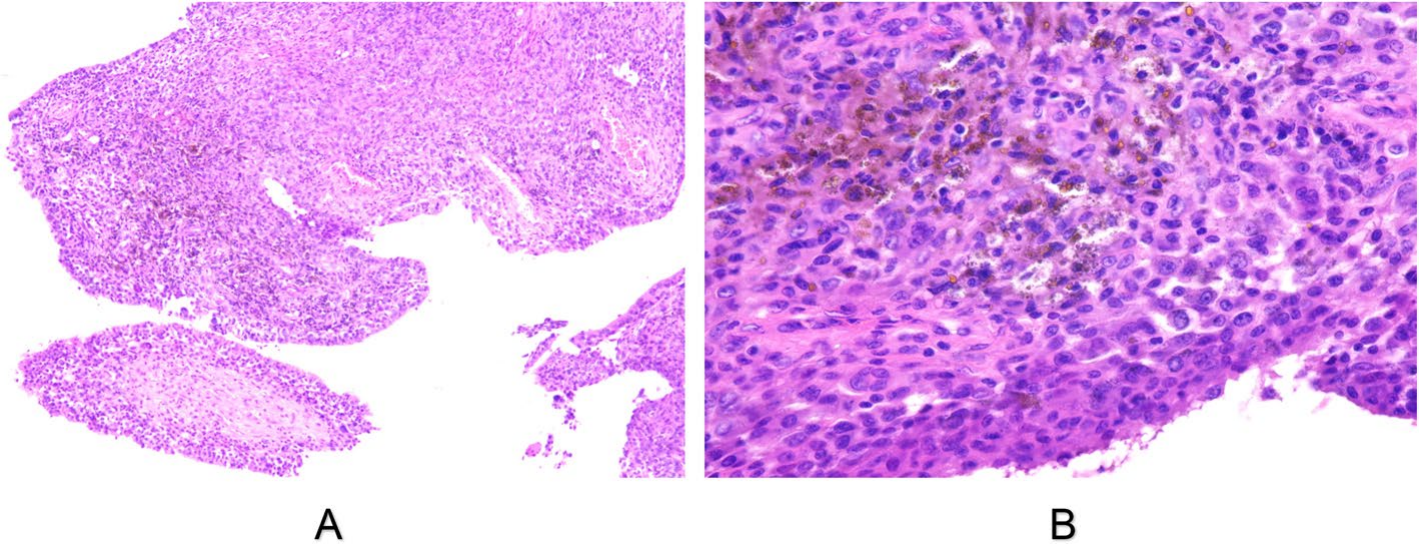
Pathological findings of the samples. **A** Photomicrograph (original magnification, × 100; hematoxylin–eosin [H-E] stain) shows villonodular fronds with overlying synovial tissue. **B** Photomicrograph (original magnification, × 400; hematoxylin–eosin [H-E] stain) shows hemosiderin-stained multinucleated giant cells

The PVNS diagnosis were confirmed in all patients by final pathologic findings (Figure 5). Histopathologic specimens obtained from the operations had papillary projections of synovial tissue accompanied with hyperplasia. Infiltration of monocytes, plasma cells, lymphocytes were seen. The lesions were composed of matted villi in which thin-walled vascular channels could be seen. The supporting stroma were packed with polyhedral stromal cells, multinucleate giant cells and macrophages.

### Functional scores

The functional scores at both first visit and final follow-up were normally distributed. All patients achieved MCID in Constant score and UCLA score improvement except case 6. When all the patients were included in the analysis, statistically significant improvements were detected in all the patient-reported outcomes (PROs) except ASES score. After case 6 was excluded, whose rotator cuff tear was irreparable, significant improvements were found in all PROs including Constant score, VAS, UCLA score and ASES score from pre-surgery to the study endpoint. The clinical outcomes were summarized in Table 2.

**Table 2:** Results of functional scores

Case No		1	2	3	4	5	6
Age at surgery, y		45	30	65	62	38	64
Times of surgeries		2	1	1	1	1	1
Procedure, Side		AS + RCR^a^, R	AS, L	AS + RCR, R	AS + RCR, R	AS, R	AS, R
VAS	Pre-op	4	3	5	3	6	3
	Final follow-up	3	0	0	0	0	4
	Results with case 6	MD, 2.83; 95% CI, 0.14 to 5.52; *P* = 0.042					
	Results without case 6	MD, 3.60; 95% CI, 1.18 to 6.02; *P* = 0.014					
Constant score	Pre-op	46	74	43	81	65	42
	Final follow-up	91	85	100	100	100	42
	Results with case 6	MD, 27.83; 95% CI, 5.17 to 50.50; *P* = 0.025					
	Results without case 6	MD, 33.40; 95% CI, 10.14 to 56.66; *P* = 0.016					
ASES score	Pre-op	57.2	85.8	71.2	85.8	57.4	85.8
	Final follow-up	92.8	100.0	100.0	100.0	100.0	57.4
	Results with case 6	MD, 17.83; 95% CI, -8.77 to 44.44; *P* = 0.145					
	Results without case 6	MD, 27.08; 95% CI, 11.27 to 42.89; *P* = 0.012					
UCLA score	Pre-op	20	25	9	24	15	17
	Final follow-up	28	35	35	35	35	11
	Results with case 6	MD, 11.67; 95% CI, 0.20 to 23.14; *P* = 0.047					
	Results without case 6	MD, 15.20; 95% CI, 5.93 to 24.47; *P* = 0.010					

*VAS* Visual Analogue Scale, *ASES* American Shoulder and Elbow Surgeons, *UCLA* University of California, Los Angeles, *Pre-op* pre-operation, *AS* arthroscopic synovectomy, *RCR* rotator cuff repair, *L* left, *R* right, *MD* mean difference, *CI* confidence interval

^a^ RCR was performed during the third surgery

### Recurrence and complications

Among the 6 included patients, one reported recurrence of shoulder PVNS (case 1). She underwent two more arthroscopic surgeries after the first operation and the disease progressed from localized PVNS to diffuse PVNS at recurrence.

**Fig. 6:**
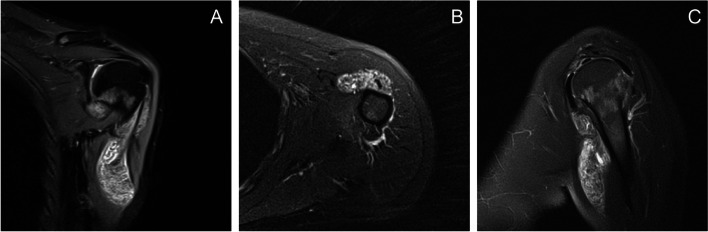
Preoperational MRI shows the massive lesion around the tendon of LHB and infiltration out of shoulder joint. **A**. The lesion on coronal section; **B**. The lesion on transverse section; **C**. The lesion on sagittal section. MRI, magnetic resonance imaging; LHB, long head of biceps

Two patients suffered from post-operative complications after the surgeries. Case 1 presented finger numb after the second surgery and the reason may be that brachial plexus was injured during the resection process when we used grasp removed a lesion in axillary capsule region that infiltrated out of axillary capsule. Fortunately, the symptom relieved after approximately 3 months. She stated that the symptom did not significantly disturb her daily life. Case number 2 suffered from rupture of the biceps brachii due to the inevitable injury of long head of biceps (LHB) during the surgery. It was found that the lesion around the tendon of LHB which was described in the operative findings part had invaded into the tendon so that part of the LHB had to be removed(Figure 6) . Fortunately, she did not get a Popeye sign. The patient was treated with conservative treatment in another hospital and got satisfactory results.

## Discussion

PVNS is a rare benign tumor characterized by synovial proliferation [5]. Shoulder PVNS is rather rarer [2]. This retrospective case series proved the low ratio of shoulder PVNS in all PVNS cases, which is as low as 2.6%. Furthermore, by reviewing the medical records of these patients, the clinical outcome of the arthroscopic synovectomy was relatively satisfying based on this study.

Despite the rarity and insignificant metastatic potential, shoulder PVNS can lead to rapid destruction of cartilage and bone and may lead to subluxation and pseudo-paralysis of the affected shoulder [14, 15]. Currently, the mainstream treatments include open and arthroscopic synovectomy [5]. Meanwhile, patients who have shoulder PVNS usually have concomitant rotator cuff tear as well [1]. This is possibly caused by the proliferating nature of PVNS, which can aggravate the degeneration of the tendons. The results of this study demonstrate that arthroscopic synovectomy combined with rotator cuff repair if needed could provide a statistically significant improvement of the patient reported outcomes in patients with shoulder PVNS. What is more, most of the improvements in postoperative scores met or exceeded MCID, which means substantial improvements in patients’ quality of life were acquired.

As early as 2001, Mahieu et al. [16] reported 2 cases of shoulder PVNS and concluded that arthroscopic synovectomy was at least as effective for synovectomy compared with open surgery. Gumina et al. [1] conducted a study in which patients with shoulder PVNS combined with massive rotator cuff tear was treated by arthroscopic synovectomy and debridement and they concluded that coexistence of irreparable rotator cuff tear may result in poor functional outcomes. In this study, 4 patients in total had rotator cuff tear and 3 of them received rotator cuff repair. All these 3 patients achieved satisfactory recovery, while the one who received isolated arthroscopic synovectomy got worse PROs after the surgery, implying that the repair is the right option when it could be done.

Using MCID as an assessment tool is one of the strengths of this study. Chiang et al. [17] conducted a retrospective case series in 2009. Like this study, the data of 5 patients with shoulder PVNS were retrospectively collected. They reported that all these patients gained symptomatic and limited functional improvement [17]. However, they did not take MCID into consideration. The study population should be taken into consideration when applying MCID [18, 19]. Since no previous study has decided the MCID in arthroscopic synovectomy of shoulder joint, we used the standard for this study, which is reasonable because of the close association between shoulder PVNS with rotator cuff tear [1]. In this research, the differences of patient-reported outcomes reach MCID in all of the patients except that of ASES score and VAS. The reason for the exception of ASES score may be that the it is a questionnaire focusing on shoulder dislocation and function of shoulder only accounts for 28% of it. The VAS improvement of patient No. 1 did not reach MCID may be due to the repeated surgeries and failed full repair after several times synovectomy so that the remaining tendon quality was not good. In a word, the improvement that arthroscopic synovectomy provides is probably clinically significant based on the results of this study.

Postoperative complications in this study include finger numb (case 1) and rupture of LHB (case 2). The appearance of these complications can be explained by the location of the lesion. Part of the first patient’s lesion located around brachial plexus in axillary fossa and the nerve might be injured during the surgery. The lesion of the second patient invaded the LHB and half of the LHB was resected, which weakened the tendon and increased the risk of tendon rupture.

There has been very little literature discussing shoulder PVNS since it is rarely seen and most studies were reported in the form of case report. Compared with open procedures, arthroscopic procedures have certain advantages including less bleeding, less trauma and shorter recovery period. Arthroscopic synovectomy has been proved to be non-inferior to open synovectomy in hip PVNS [20]. Whether it is a viable option in shoulder PVNS is still worth exploring. Pereira et al. [2] reported a case of shoulder PVNS involved subacromial bursa, in which an arthroscopic synovectomy was performed to totally remove the synovium, fluid and loose bodies and the patient got satisfactorily recovered, that is comparable to this study. However, they did not report any specific outcomes such as functional scores of the patient. Serra et al. [21] reported a case of diffuse shoulder PVNS. The patient underwent an arthroscopic near-total synovectomy and got pain relief and increase of range of motion. However, because of the incomplete synovial resection, the patient received further radiotherapy and presented free of symptoms at 1-month follow-up. Nevertheless, the patient refused further follow-up so that the outcome of a long-term follow-up is unavailable. To our knowledge, although only 5 patients and 8 surgeries were enrolled, this study is still one of the studies with the most cases of shoulder PVNS compared to previous case reports and case series focusing on shoulder PVNS [1, 2, 14, 15, 17, 21–23]. Furthermore, the mean follow-up time of this study is also one of the longest. These advantages have made this research relatively more convincing.

This study does have some limitations. Firstly, it is a retrospective case series with a small sample size from a single institution, and not all the patients underwent the exact same procedure. However, shoulder PVNS is known to be extremely rare and fewer than 40 cases have been reported based on our literature search. As a result, it is quite difficult to collect a larger number of cases who underwent the exactly same procudure in a single institution. Secondly, this is not a comparative study, so that we could not directly compare the efficacy of arthroscopic and open synovectomy. However, the 3 patients who underwent open surgery went through other concurrent procedures such as humeral head replacement and bone defect filling, making it improper to include them in this study. Further comparative researches with large sample size are still needed. Third, the effect of radiotherapy is not included in this research although it is a safe and effective treatment for PVNS after incomplete resection [24]. The reason is that with only 1 patient went through radiotherapy after the surgery, we cannot draw a firm conclusion. Future studies with more cases treated with radiotherapy will be able to provide more knowledge of it.

## Conclusion

Arthroscopic synovectomy in the setting of shoulder PVNS can improve patients’ function. A concurrent rotator cuff repair is recommended if it is needed.

## Data Availability

The datasets generated and/or analysed during the current study are not publicly available due to privacy restrictions, but are available from the corresponding author on reasonable request.

## Abbreviations

PVNS: Pigmented villonodular synovitis
MRI: Magnetic resonance imaging
BMI: Body mass index
HE: Hematoxylin and eosin
VAS: Visual Analogue Scale
UCLA: University of California
ASES: American Shoulder and Elbow Surgeons
MCID: Minimal clinically important difference
PRO: Patient-reported outcome
MD: Mean difference
CI: Confidence interval
LHB: Long head of biceps

## References

[1] Gumina S, Carbone S, Campagna V, Castagna A, Della Rocca C, Rocca CD (2013). Pigmented villonodular synovitis of the shoulder associated with massive rotator cuff tear treated by arthroscopic synovectomy and debridement. Musculoskelet Surg.

[2] Pereira VL, Baldan AR, Andreoli CV, Belangero PS, de Castro Pochini A, Ejnisman B (2021). Subacromial pigmented villonodular synovitis: case report and review. J Surg Case Rep.

[3] Nazal MR, Parsa A, Gibbs JS, Abraham PF, Martin SD (2020). Mid-Term Results of Arthroscopic Synovectomy for Pigmented Villonodular Synovitis of the Hip. Arthrosc J Arthrosc Relat Surg Off Publ Arthrosc Assoc N Am Int Arthrosc Assoc.

[4] Bernthal NM, Ishmael CR, Burke ZDC (2020). Management of Pigmented Villonodular Synovitis (PVNS): an Orthopedic Surgeon’s Perspective. Curr Oncol Rep.

[5] Noailles T, Brulefert K, Briand S, Longis P-M, Andrieu K, Chalopin A (2017). Giant cell tumor of tendon sheath: Open surgery or arthroscopic synovectomy? A systematic review of the literature. Orthop Traumatol Surg Res OTSR.

[6] Burton TM, Ye X, Parker ED, Bancroft T, Healey J. Burden of Illness Associated with Tenosynovial Giant Cell Tumors. Clin Ther. 2018;40:593–602.e1. 10.1016/j.clinthera.2018.03.001 PMC7440779 29580718

[7] Sevimli R, Alan S, Eriten S, Polat H, Turkmen E. Our clinical outcomes in patients operated with the diagnosis of pigmented villonodular synovitis. Med Sci Int Med J. 2019;1:581-5.

[8] Paxton ES, Backus J, Keener J, Brophy RH (2013). Shoulder arthroscopy: basic principles of positioning, anesthesia, and portal anatomy. J Am Acad Orthop Surg.

[9] Boutsiadis A, Chen S, Jiang C, Lenoir H, Delsol P, Barth J. Long Head of the Biceps as a Suitable Available Local Tissue Autograft for Superior Capsular Reconstruction: “The Chinese Way.” Arthrosc Tech. 2017;6:e1559–66. 10.1016/j.eats.2017.06.030 PMC5709836 29354474

[10] Lo IKY, Burkhart SS (2003). Double-row arthroscopic rotator cuff repair: re-establishing the footprint of the rotator cuff. Arthrosc J Arthrosc Relat Surg.

[11] Malavolta EA, Yamamoto GJ, Bussius DT, Assunção JH, Andrade-Silva FB, Gracitelli MEC, et al. Establishing minimal clinically important difference for the UCLA and ASES scores after rotator cuff repair. Orthop Traumatol Surg Res OTSR. 2021;108(2):102894. 10.1016/j.otsr.2021.102894 33746073

[12] Xu S, Chen JY, Lie HME, Hao Y, Lie DTT (2020). Minimal Clinically Important Difference of Oxford, Constant, and UCLA shoulder score for arthroscopic rotator cuff repair. J Orthop.

[13] Tashjian RZ, Hung M, Keener JD, Bowen RC, McAllister J, Chen W (2017). Determining the minimal clinically important difference for the American Shoulder and Elbow Surgeons score, Simple Shoulder Test, and visual analog scale (VAS) measuring pain after shoulder arthroplasty. J Shoulder Elbow Surg.

[14] Kwon M, Bang J-Y, Nam KH (2020). Rapid destruction of shoulder joint by pigmented villonodular synovitis treated by hemiarthroplasty: a case report. Int J Surg Case Rep.

[15] Lee K, Kim H, Lee H, Jang I-T, Choi S (2021). An abrupt-onset shoulder joint subluxation and pseudoparalysis caused by intraarticular pigmented villonodular synovitis: a case report. Jt Dis Relat Surg.

[16] Mahieu X, Chaouat G, Blin JL, Frank A, Hardy P (2001). Arthroscopic treatment of pigmented villonodular synovitis of the shoulder. Arthrosc J Arthrosc Relat Surg Off Publ Arthrosc Assoc N Am Int Arthrosc Assoc.

[17] Chiang E-R, Ma H-L, Wang S-T, Hung S-C, Chen T-H (2009). Arthroscopic treatment for pigmented villonodular synovitis of the shoulder associated with massive rotator cuff tear. Arthrosc J Arthrosc Relat Surg Off Publ Arthrosc Assoc N Am Int Arthrosc Assoc.

[18] Engel L, Beaton DE, Touma Z (2018). Minimal Clinically Important Difference: A Review of Outcome Measure Score Interpretation. Rheum Dis Clin North Am.

[19] Sedaghat AR (2019). Understanding the Minimal Clinically Important Difference (MCID) of Patient-Reported Outcome Measures. Otolaryngol-Head Neck Surg Off J Am Acad Otolaryngol-Head Neck Surg.

[20] Cheok T, Wills K, Berman M, Jennings MP, Poonnoose PM. Open or Arthroscopic Synovectomy Is the Preferred Management Option in Pigmented Villonodular Synovitis of the Hip Joint Without Evidence of Degeneration: A Systematic Review of 20 Studies. Arthrosc Sports Med Rehabil. 2022. 10.1016/j.asmr.2022.06.008 10.1016/j.asmr.2022.06.008 PMC9596911 36312712

[21] Serra TQ, Morais J, Gonçalves Z, Agostinho F, Melo G, Henriques M (2017). An unusual case of diffuse pigmented villonodular synovitis of the shoulder: a multidisciplinary approach with arthroscopic synovectomy and adjuvant radiotherapy. Eur J Rheumatol.

[22] Sayegh ET, Wilk RM (2021). Pigmented Villonodular Synovitis of the Glenohumeral Joint and Biceps Tendon Sheath. Cureus.

[23] Koh KH, Lim KS, Yoo JC (2010). Arthroscopic treatment of pigmented villonodular synovitis involving bilateral shoulders. Orthopedics.

[24] Heyd R, Micke O, Berger B, Eich HT, Ackermann H, Seegenschmiedt MH (2010). Radiation therapy for treatment of pigmented villonodular synovitis: results of a national patterns of care study. Int J Radiat Oncol Biol Phys.

